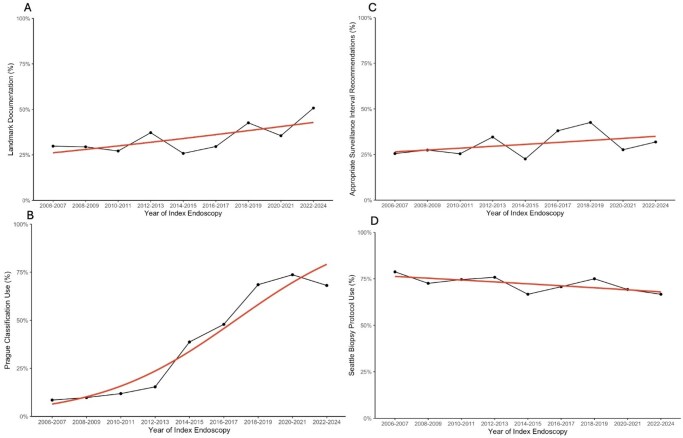# Poster Session I - A59 TEMPORAL TRENDS IN ADHERENCE TO QUALITY INDICATORS IN ENDOSCOPIC SURVEILLANCE OF BARRETT’S ESOPHAGUS

**DOI:** 10.1093/jcag/gwaf042.059

**Published:** 2026-02-13

**Authors:** Y Yao, C Roda, A Hu, S Sui, Y Mandoub, D M Koerber, A Walia, P Tavakoli, S Pang, E Taylor, D Motomura, E Lam, R A Enns, W Xiong, N C Shahidi

**Affiliations:** Department of Medicine, University of British Columbia, Vancouver, BC, Canada; Department of Medicine, University of British Columbia, Vancouver, BC, Canada; St. Paul’s Hospital, Digestive Health Centre, Vancouver, BC, Canada; St. Paul’s Hospital, Digestive Health Centre, Vancouver, BC, Canada; St. Paul’s Hospital, Digestive Health Centre, Vancouver, BC, Canada; Department of Medicine, University of British Columbia, Vancouver, BC, Canada; Department of Medicine, University of British Columbia, Vancouver, BC, Canada; St. Paul’s Hospital, Digestive Health Centre, Vancouver, BC, Canada; St. Paul’s Hospital, Digestive Health Centre, Vancouver, BC, Canada; St. Paul’s Hospital, Digestive Health Centre, Vancouver, BC, Canada; St. Paul’s Hospital, Digestive Health Centre, Vancouver, BC, Canada; St. Paul’s Hospital, Digestive Health Centre, Vancouver, BC, Canada; St. Paul’s Hospital, Digestive Health Centre, Vancouver, BC, Canada; Department of Pathology and Laboratory Medicine, University of British Columbia, Vancouver, BC, Canada; St. Paul’s Hospital, Digestive Health Centre, Vancouver, BC, Canada

## Abstract

**Background:**

Endoscopic surveillance of Barrett’s Esophagus (BE) enables timely endotherapy to prevent progression to esophageal adenocarcinoma (EAC). Guidelines emphasize using quality indicators (QIs) to optimize outcomes, yet adherence remains inconsistent.

**Aims:**

To evaluate temporal trends in adherence to four QIs: key anatomic landmark documentation, Prague classification (PC) use, Seattle biopsy protocol (SP) use, and guideline surveillance interval recommendations.

**Methods:**

From 01/2006 - 12/2024, 869 patients with histologically confirmed intestinal metaplasia with goblet cells were identified from a validated pathology registry at a tertiary care center. Patients with BE (>1 cm of columnar epithelium above the gastric folds on index endoscopy) underwent retrospective chart review. Adherence to all QIs was determined from the index endoscopy report, except SP use, which was assessed from the first surveillance pathology report. Logistic regression assessed predictors of QI adherence by year of index endoscopy. A composite adherence score was calculated as the sum of adhered QIs. Significance was set at p < 0.05.

**Results:**

Patients with confirmed dysplasia, EAC or indefinite findings on index endoscopy (n = 97), and those referred for neoplastic management (n = 174) were excluded. 598 patients (465 male, 133 female) were included. Overall QI adherence was low: 34.1% had complete landmark documentation, 39.8% used PC, and 30.4% recommended appropriate surveillance intervals. Among 396 patients with surveillance endoscopy, only 73% used the SP. Median composite adherence score was 2 (IQR 1). Adherence to PC increased over time; patients in 2022-2024 had significantly higher odds of adherence than 2006-2007 (OR 23.4, 95% CI 8.24–85.6, p < 0.001). Adherence to other QIs did not significantly vary over time. Compared to general endoscopists, interventional endoscopists had higher adherence to PC (59.4% vs. 34.5%, p < 0.001) and guideline surveillance interval recommendations (41.4% vs. 27.4%, p < 0.05).

**Conclusions:**

Adherence to BE QIs remains suboptimal, with only PC use improving over time. Interventional endoscopists show higher adherence to selected QIs, underscoring opportunities for initiatives to standardize BE surveillance practices.

A59 Table 1:

**Funding Agencies:**

Neal Shahidi is supported by the Michael Smith Health Research BC Scholar Award.